# Emphysematous Pyelonephritis due to *Candida glabrata* and *Candida tropicalis* Bezoar: A Case Report

**DOI:** 10.1155/crdi/2502643

**Published:** 2026-08-05

**Authors:** John Corbyn Cravero, Hunter Martin, Lauren Sisco

**Affiliations:** ^1^ Department of Internal Medicine, Baylor Scott & White Medical Center, Temple, Texas, USA; ^2^ Department of Infectious Disease, Baylor Scott & White Medical Center, Temple, Texas, USA

**Keywords:** amphotericin B, *Candida*, flucytosine, percutaneous nephrostomy, urinary fungal bezoar

## Abstract

Emphysematous pyelonephritis (EPN) is a severe, necrotizing infection that can affect the renal parenchyma, collecting system, or perinephric tissue. Although typically caused by enteric Gram‐negative bacteria, infections secondary to *Candida* occur rarely. We present the case of a 72‐year‐old female who was transferred from an outside hospital with a chief complaint of abdominal pain in the setting of dislodged percutaneous nephrostomy tube for chronic hydronephrosis. Clinically, she presented without evidence of septic shock but was found to have elevated creatinine (6.04), concerning for acute renal failure. Urology was consulted and performed cystoscopy that showed a large bilateral mass consistent with fungal ball formation. Subsequent urine cultures speciated *Candida tropicalis* and *Candida glabrata*. Per infectious disease recommendations, the patient was initially started on treatment with fluconazole and flucytosine; however, fluconazole was switched to amphotericin B deoxycholate after sensitivities reported resistance to fluconazole for *Candida tropicalis*. Additionally, urology performed a bilateral percutaneous nephroscopy with ultrasonic lithotripsy for removal of her fungal bezoar on Day 22 of her hospitalization with subsequent nephrostograms that showed a patent genitourinary system. This case demonstrates successful treatment of EPN secondary to azole‐resistant *Candida* species using combined systemic antifungal therapy and an endourologic approach. This avoided the use of percutaneous nephrostomy irrigation with amphotericin deoxycholate as recommended in current guidelines and prevented potential complications this procedure carries.

**Key Messages**
• Emphysematous pyelonephritis is a severe, necrotizing infection that can affect the renal parenchyma, collecting system, or perinephric tissue and is typically caused by enteric Gram‐negative bacteria or more rarely *Candida*.
• The current Infectious Disease Society of America (IDSA) guideline recommends percutaneous nephrostomy irrigation with amphotericin B deoxycholate in patients with genitourinary fungal balls.
• Our case report demonstrates successful management with endourologic intervention and systemic flucytosine, avoiding local irrigation of amphotericin b deoxycholate.

## 1. Introduction

Emphysematous pyelonephritis (EPN) is a severe, necrotizing infection that results in gas formation within the renal parenchyma, collecting system, or perinephric tissue [1]. While the majority of EPN cases are caused by enteric Gram‐negative bacteria, such as *Escherichia coli* (49%–67%) and *Klebsiella pneumoniae* (20%–24%), and *Proteus* species (5%–18%), infections due to Candida are relatively rare [2]. Diabetes is the most significant risk factor and occurs in 90% of EPN cases, followed by obstructive uropathy (29%–49%) [2, 3]. Mortality ranges from 20%–80% but is mainly attributed to complications from sepsis [3].

The incidence and prevalence of *Candida* bezoars are currently unknown, and cases of EPN in the setting of obstructive *Candida* bezoars are exceedingly rare [4]. Risk factors for the development of Candida bezoars include anatomic urinary tract abnormalities, diabetes mellitus, indwelling Foley catheters, and an increased use of broad‐spectrum antibiotics [4]. We present a case of EPN secondary to *Candida glabrata* and *Candida tropicalis* in an elderly female with diabetes mellitus that responded to treatment with intravenous infusions of amphotericin and flucytosine.

## 2. Case Presentation

The patient was a 72‐year‐old female with Type 2 diabetes mellitus, Stage 3b chronic kidney disease with chronic hydronephrosis status postbilateral percutaneous nephrostomy tube placement, vaginal prolapse with pessary placement, and hypertension who presented with a chief complaint of abdominal pain in the setting of a dislodged percutaneous nephrostomy tube. Two days prior to this admission, the patient had placement of bilateral ureteral stents and bilateral percutaneous nephrostomy tubes for postobstructive kidney injury and EPN. However, repeat CT imaging of the abdomen performed the following day showed displacement of the bilateral ureteral stents. Laboratory studies at this time were significant for leukocytosis (17.45k). Based on these findings, the patient was started on piperacillin–tazobactam antibiotics, and consultation was sought with infectious disease, nephrology, and urology.

**FIGURE 1:**
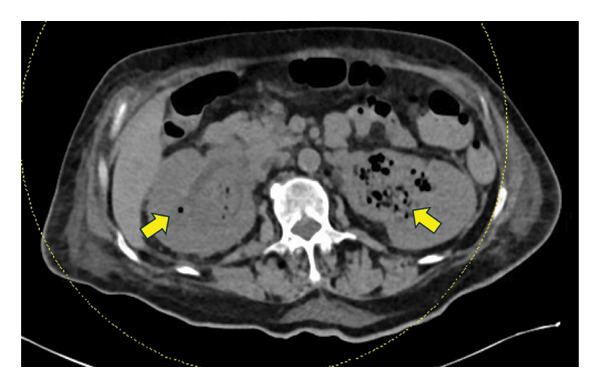
Noncontrast computed tomography (CT) abdomen and pelvis axial view, demonstrating severe dilation of the left and right collecting systems with fluid and gas as indicated by yellow arrows.

**FIGURE 2:**
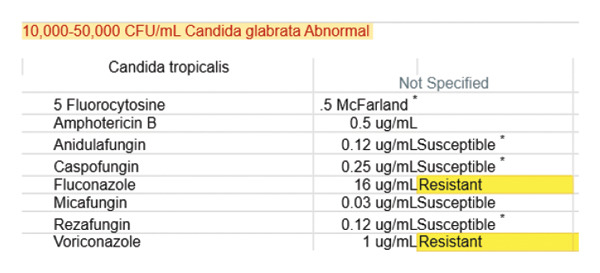
Sensitivity data for *Candida tropicalis*. Note: *Candida glabrata* data are not reported due to inability to isolate this species from *Candida tropicalis.*

On hospital day 1, the patient was admitted to our facility to a med‐surge floor without hemodynamic instability or evidence of shock. A repeat CT abdomen/pelvis was notable for EPN with moderate to severe right‐sided hydronephrosis (Figure 1). Laboratory studies were significant for an elevated creatinine (6.04) from a baseline of 0.7–0.8, leukocytosis of 16.8, and multiple electrolyte derangements in the setting of renal failure (Table 1). Piperacillin–tazobactam was de‐escalated to ceftriaxone based on prior cultures. On hospital day 4, the patient underwent a bilateral nephrostomy tube exchange. On hospital day 6, urology removed her bilateral ureteral stents and performed a nephrostogram that showed the presence of a large bilateral mass with complete right‐sided obstruction. Urine cultures collected on hospital day 1 from the left nephrostomy tube were positive for *Candida tropicalis* (10–50k) and *Candida glabrata* (10–50k). Ceftriaxone was discontinued. Blood cultures collected on hospital day 1 were negative for growth. Prior to antifungal sensitivity testing, infectious disease recommended initial therapy with renally dosed fluconazole 100 mg that was increased to fluconazole 200 mg daily on hospital day 8. An additional right‐sided percutaneous nephrostomy tube was placed due to decreased urinary output and in preparation for irrigation; however, irrigation was ultimately deferred based on a risk–benefit discussion with the urology staff who expressed concern about possible seeding of the peritoneum with irrigation and lack of a standardized protocol. On hospital day 12, flucytosine 25 mg/kg was added per infectious disease recommendations pending sensitivities for *Candida* species, which ultimately showed *Candida tropicalis* resistant to fluconazole and voriconazole (Figure 2). Subsequent sensitivities for *Candida glabrata* were not reported due to the microbiology laboratory’s inability to isolate *Candida glabrata* from *Candida tropicalis*. Based on antifungal sensitivity data and concern for intrinsic azole resistance associated with *Candida glabrata*, fluconazole was discontinued, and amphotericin B deoxycholate 0.3 mg/kg was initiated on hospital day 13.

**TABLE 1:** Patient laboratory data.

Laboratory	Start of admission (hospital day 2)	Discharge (hospital day 30)
Sodium (meQ/L)	132	132
Potassium (meQ/L)	5.5	5.0
Chloride (meQ/L)	107	105
BUN (mg/dL)	59	39
Creatinine (mg/dL)	6.04^∗^	1.84
Phosphorus (mg/dL)	7.0	3.9
Alkaline phosphatase (U/L)	220	298
Aspartate aminotransferase (AST) (U/L)	23	17
Alanine aminotransferase (ALT) (U/L)	< 6	8
White blood cell count (10^3^/μL)	16.8	16.2
Hematocrit	7.9/24.6	9.9/31.1

^∗^Denotes the maximum/peak value during admission.

**FIGURE 3:**
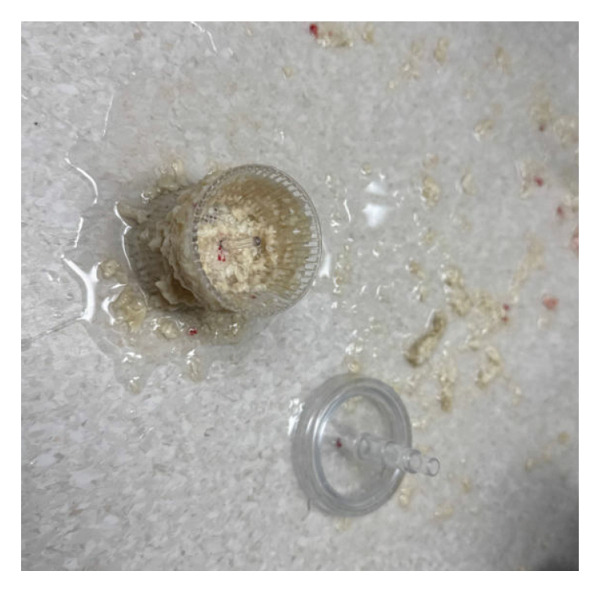
Aspirated candidal bezoar, showing the aspirated candidal bezoar obtained during a bilateral percutaneous nephroscopy with ultrasonic lithotripsy.

**FIGURE 4:**
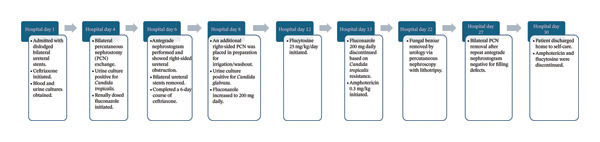
Timeline of hospitalization.

While on combined therapy with amphotericin and flucytosine, urology performed a bilateral percutaneous nephroscopy with ultrasonic lithotripsy for removal of her fungal bezoar (Figure 3) on hospital day 22. On hospital day 26, urology performed a repeat bilateral nephrostogram that showed patent ureters, which allowed for removal of her percutaneous nephrostomy tubes. She continued dual coverage with amphotericin and flucytosine for 7 days from this final urologic procedure. On hospital day 30, her bilateral percutaneous nephrostomy tubes were removed, amphotericin and flucytosine were discontinued, and she was successfully discharged home. She was noted to have a persistently elevated white blood cell count on the day of discharge; however, this was felt to be clinically insignificant due to her overall improved clinical presentation and lack of other findings that suggested an alternative systemic process or infection. A complete timeline of these events is represented in Figure 4. While the patient was discharged home in a stable condition, she failed to show up for follow‐up with infectious disease, nephrology, and urology and did not undergo any repeat laboratory or imaging studies.

## 3. Discussion

This patient’s case represents successful treatment of candidal EPN with endoscopic source control, thereby avoiding nephrectomy and genitourinary tract irrigation. The most recent Infectious Diseases Society of America (IDSA) guidelines on candidiasis propose a three‐pronged approach to the treatment of candidal urinary tract infections associated with fungal balls: (1) surgical or endoscopic removal of the obstructing mycelial mass; (2) antifungal treatment with fluconazole (drug of choice), flucytosine, or amphotericin deoxycholate; and (3) irrigation through nephrostomy tubes, if present, with amphotericin deoxycholate (25–50 mg in 200–500 mL of sterile water). [5]. Regarding endoscopic removal, the IDSA references anecdotal reports largely based on pediatric cases. One citation by Shih et al. from 2005 describes a case of percutaneous removal of mycetoma in a 4‐month‐old infant who presented with renal failure [6]. Another report by Babu and Hutton from 2004 describes treatment with streptokinase in a low‐birth‐weight infant with renal fungal balls and pelvic–ureteric obstruction [7]. Our case expands this body of literature by showing successful treatment with the use of endoscopic source control in an adult patient.

Review of the medical literature shows several case reports similar to ours that utilized a combined approach with systemic therapy and endourologic intervention [8–14]. Additionally, there are also a handful of case reports that describe percutaneous nephrostomy irrigation with antifungals as successful treatment of obstructive genitourinary fungal bezoars, which is in accordance with IDSA guidelines [15, 16]. Table 2 summarizes these case reports in terms of patient demographics, the candidal species involved, and the treatment approach. Based on these results, successful treatment occurred in adult patients whose ages ranged from 35 to 80 and had multiple comorbidities, including diabetes mellitus, cancer, and immunosuppression. Treatment was successful for various species of *Candida*, including *C. sake, C. albicans, C. tropicalis*, and *C. glabrata*. More importantly, all patients received systemic antifungal medications and urologic intervention, either with local PCN irrigation and/or endoscopic bezoar removal. None of these patients had evidence of recurrence at the time of follow‐up, which ranged from 1 week to 20 months.

**TABLE 2:** Summation of case reports involving *Candida* bezoars.

Author (year published)	Demographic information (age, sex) and relevant medical comorbidities	Candidal species	Medical therapy treatment	Local/PCN irrigation therapy	Endoscopic therapy	Outcome
Arend et al. (2006)	72‐year‐old male ‐ Renal transplant	*Candida sake*	IV caspofungin	Local irrigation with amphotericin B and caspofungin	Cysto‐pyelostomy	No recurrence of fungal infection during 20‐month post‐op follow‐up.

Davis et al. (2013)	80‐year‐old female ‐ Immunosuppressed on treatment for RA	*Candida albicans*	IV caspofungin	Rigid ureteroscopy with transurethral resection of the left ureteric orifice and distal intravesical ureter	NA	Well‐healed, patent left ureteric orifice without recurrence of fungal infection at 1‐month follow‐up.

Abdeljaleel et al. (2018)	60‐year‐old male ‐ Diabetes mellitus ‐ Refractory urinary retention	*Candida albicans*	IV fluconazole Anidulafungin	Local irrigation with fluconazole	NA	Normal follow‐up renal ultrasound and negative repeat urine culture at 1‐week follow‐up.

Abuelnaga et al. (2019)	80‐year‐old diabetes mellitus ‐ History of neo‐adjuvant long course chemo‐radiotherapy followed by resection and loop ileostomy for a rectal tumor.	*Candida albicans*	IV fluconazole	NA	Flexible ureteroscopy	No recurrence of fungal infection based on flexible ureteroscopy at 2‐week follow‐up.

Vishwambaram et al. (2020)	65‐year‐old female ‐ Recurrent fungal cystitis	*Candida glabrata*	IV liposomal amphotericin B and oral flucytosine	Local irrigation with amphotericin B deoxycholate	NA	No recurrence of fungal infection at 6‐ and 14‐week follow‐up.

Zhang et al. (2023)	67‐year‐old female ‐ Diabetes mellitus ‐ Hypertension	*Candida tropicalis*	‐ Antifungal medications ‐ Antibacterial medications		Percutaneous nephroscopic fungal ball removal.	No recurrence of fungal infection at 1‐year follow‐up.

Villegas et al. (2024)	46‐year‐old female ‐ Diabetes mellitus ‐ Nephrolithiasis	*Candida glabrata*	IV micafungin	Retrograde irrigation with amphotericin B	NA	No recurrence of fungal infection during follow‐up.

Cark and Gaunt (1999)	35‐year‐old female ‐ Cervical squamous cell carcinoma treated with radiation therapy and chemotherapy	*Candida albicans*	Systemic fluconazole	Local irrigation with fluconazole	NA	Patient died at 6‐month follow‐up; no evidence of fungal recurrence at the time of death.

Chung et al. (2001)	57‐year‐old female ‐ Diabetes mellitus ‐ ESRD on hemodialysis	*Candida* spp.	Oral fluconazole	Ureteral catheter irrigation with fluconazole	NA	Urine cultures on Day 10 and Week 4 of treatment showed no growth of *Candida* spp.

Despite similarities to previously reported cases, our report has several important limitations. First, antifungal susceptibility testing was not performed for the *Candida glabrata* isolate because the microbiology laboratory was unable to separate *Candida glabrata* from the coisolated *Candida tropicalis*. Successful eradication of invasive candidal infections depends heavily on appropriate antifungal selection, particularly for species with known intrinsic or acquired resistance patterns such as *Candida glabrata*. Although treatment with flucytosine and amphotericin B deoxycholate was guided by the available susceptibility data, the likelihood of treatment failure with this regimen is considered low based on existing microbiologic evidence and established antifungal activity against *Candida glabrata*.

Second, genitourinary irrigation through the nephrostomy tubes was not pursued following a multidisciplinary risk–benefit assessment with the urology service due to concerns that the procedure could exacerbate peritoneal contamination and worsen the patient’s infection. Whether this intervention would have altered the clinical outcome remains uncertain. This question is further complicated by a third limitation: the patient’s lack of outpatient follow‐up after hospital discharge, which precluded assessment of her long‐term clinical response and overall outcome.

## 4. Conclusion

EPN secondary to candidal infections is a rare occurrence. This case demonstrates successful treatment with systemic flucytosine and amphotericin B and endourologic ultrasonic lithotripsy without the need for local antifungal irrigation via percutaneous nephrostomies in a patient with EPN secondary to multidrug‐resistant *Candida* infections. This case and case review also adds to the growing body of literature on this subject and underscores the need for future studies to affirm guidelines that emphasize this combined approach with systemic therapy and endourologic source control in the treatment of candidal bezoar infections.

## Author Contributions

Dr. Hunter Martin was responsible for the case discussion. Dr. John Corbyn Cravero was responsible for the introduction, discussion, and conclusion. Dr. Lauren Sisco was our attending staff and oversaw all aspects of the case report.

## Funding

The authors of this manuscript do not have any funding to declare. All the authors are currently under the employment of the Baylor Scott & White Healthcare system.

## Disclosure

The manuscript and the case described have not been presented at a conference or published elsewhere.

## Ethics Statement

The authors of this paper maintain their professional and academic integrity throughout the process of composing this manuscript. This retrospective review of patient data did not require ethical approval in accordance with local/national guidelines. Written informed consent was obtained from the patient for publication of the details of her medical case and any accompanying images.

## Consent

Written consent from the patient was obtained and is available upon request. IRB approval was not needed.

## Conflicts of Interest

The authors declare no conflicts of interest.

## Data Availability

No new data were generated.
